# Burden of Culture Confirmed Enteric Fever Cases in Karachi, Pakistan: Surveillance For Enteric Fever in Asia Project (SEAP), 2016–2019

**DOI:** 10.1093/cid/ciaa1308

**Published:** 2020-12-01

**Authors:** Mohammad T Yousafzai, Seema Irfan, Rozina S Thobani, Abdul M Kazi, Aneeta Hotwani, Ashraf M Memon, Khalid Iqbal, Saqib H Qazi, Nasir S Saddal, Najeeb Rahman, Irum F Dehraj, Mohammed J Hunzai, Junaid Mehmood, Denise O Garrett, Samir K Saha, Jason R Andrews, Stephen P Luby, Farah N Qamar

**Affiliations:** 1 Department of Pediatrics and Child Health, Aga Khan University, Karachi, Pakistan; 2 Department of Pathology and Laboratory Medicine, Aga Khan University, Karachi, Pakistan; 3 Clinical Laboratory, Kharadar General Hospital, Karachi, Pakistan; 4 Department of Surgery, Aga Khan University, Karachi, Pakistan; 5 National Institute of Child Health, Karachi, Pakistan; 6 Applied Epidemiology, Sabin Vaccine Institute, Washington, DC, USA; 7 Child Health Research Foundation, Department of Microbiology, Dhaka Shishu (Children) Hospital, Bangladesh; 8 Infectious Diseases and Geographic Medicine, Stanford University, Stanford, California, USA

**Keywords:** Enteric fever, burden, *Salmonella* Typhi, typhoid fever, Pakistan

## Abstract

**Background:**

The Surveillance for Enteric Fever in Asia Project (SEAP) is a multicenter, multicountry study conducted in Pakistan, Nepal, and Bangladesh. The objectives of the study were to characterize disease incidence among patients with enteric fever. We report the burden of enteric fever at selected sites of Karachi, Pakistan.

**Methods:**

During September 2016 to September 2019, prospective surveillance was conducted at inpatient, outpatient, surgical departments, and laboratory networks of Aga Khan University Hospital, Kharadar General Hospital, and surgery units of National Institute of Child Health and Jinnah Postgraduate Medical Centre. Socio-demographic, clinical, and laboratory data were obtained from all suspected or confirmed enteric fever cases.

**Results:**

Overall, 22% (2230/10 094) of patients enrolled were culture-positive for enteric fever. 94% (2093/2230) of isolates were *Salmonella* Typhi and 6% (137/2230) were *S*. Paratyphi. 15% of isolates multi-drug resistant (MDR) to first-line antibiotics and 60% were extensively drug-resistant (XDR), resistant to first-line antibiotics, fluoroquinolones and third generation cephalosporin.

**Conclusion:**

Enteric fever cases have increased during the last 3 years with large proportion of drug resistant *S.* Typhi cases. However, the burden of paratyphoid is still relatively low. Strengthening the existing surveillance system for enteric fever and antimicrobial resistance at the national level is recommended in Pakistan to inform prevention measures. While typhoid vaccination can significantly decrease the burden of typhoid and may also impact antimicrobial resistance, water, sanitation, and hygiene improvement is highly recommended to prevent the spread of enteric fever.

Typhoid fever and paratyphoid fever are caused by infection with *Salmonella enterica* subspecies serovars Typhi (*S*. Typhi) and (*S*. Paratyphi). Both are generally termed as enteric fever. Globally, 14.3 million cases of typhoid and paratyphoid occurred in 2017, resulting in 135.9 thousand deaths with a substantial burden (69.6%) in South Asia [1]. Enteric fever is endemic in low resource countries in South Asia, including Pakistan, due to limited access to safe drinking water and poor sanitation and hygiene [2, 3]. It is the most common bacteremic illness in children in Pakistan, with rates as high as 1000 cases per 100 000 child-years having been reported from Karachi [4]. According to a recent study, only 20% of the population in Pakistan has access to safe drinking water and the remaining 80% of the population are compelled to use unsafe drinking water due to the scarcity of safe and healthy drinking water sources [5]. In Pakistan, typhoid perforation remains a frequently fatal disease with high prevalence in remote areas of Sindh Province [6]. The Surveillance for Enteric Fever in Asia Project (SEAP) was a comprehensive, multi-country, and multisite study in South Asia. SEAP Phase I, 2012–2015, was a retrospective surveillance study initiated to inform data collection for prospective surveillance and to capture clinical aspects of the disease. The study provided retrospective evidence on burden, illness severity, and antimicrobial resistance (AMR) trends. In that study, the highest proportion (52%) of hospitalized *S*. Typhi cases occurred among children aged 5–15 years, and multidrug resistance (MDR) was found in 52% of *S.* Typhi isolates [7, 8]. SEAP Phase II (2016–2019) was a prospective surveillance study, established to fill specific data gaps linked to burden, age distribution, antimicrobial resistance (AMR), severity and complications of the disease, and cost of illness.

It was established to estimate the burden of enteric fever utilizing a hybrid approach that involved pairing facility-based surveillance with population-based healthcare utilization studies [8].

Findings from this urban, high endemicity area of Karachi, Pakistan, could be used by the government policymakers and Gavi, the Vaccine Alliance, to advocate for the introduction of the typhoid conjugate vaccine (TCV) in the routine immunization program of Pakistan. Here, we report the burden of enteric fever in Karachi, Pakistan, including the distribution of *S*. Typhi and *S*. Paratyphi by age groups, seasonality, care-seeking behavior, complications among antibiotic-resistant and nonresistant cases in Karachi, Pakistan.

## METHODS

### Study Design and Setting

**Figure 1.**
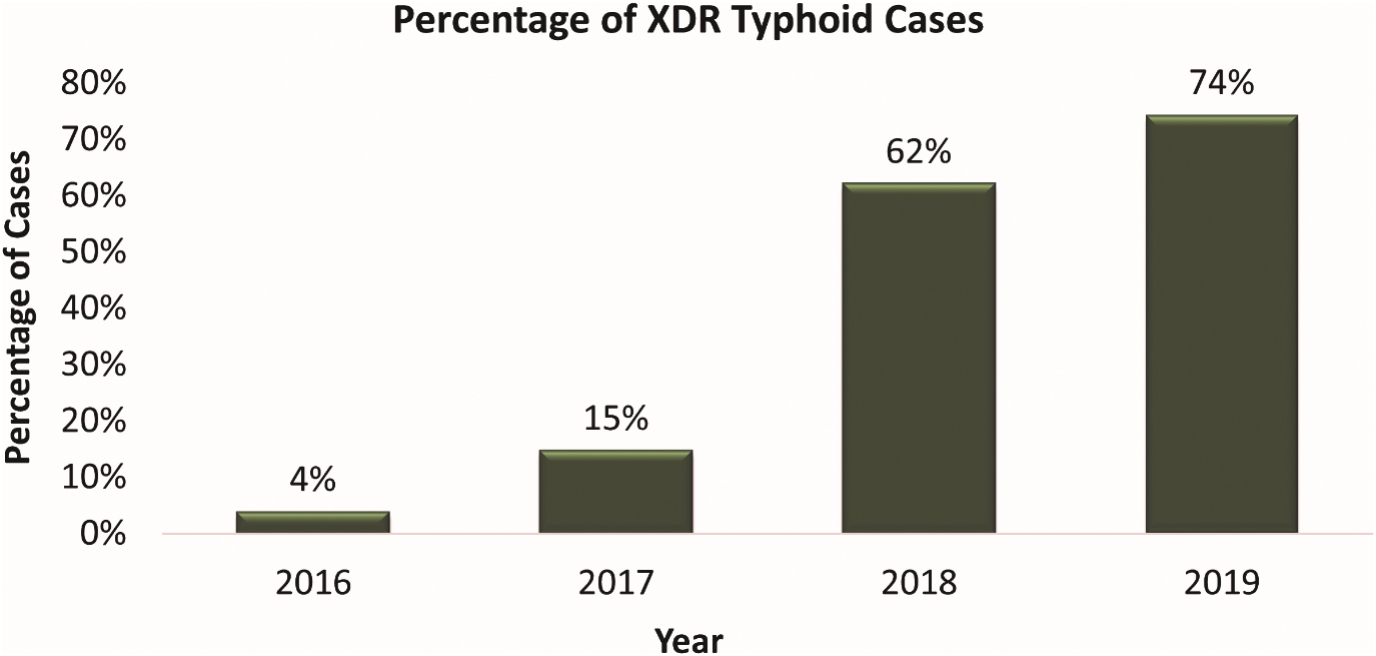
Map of the SEAP study sites in Karachi, Pakistan, 2016–2019.

We conducted this prospective surveillance study at inpatient, outpatient, surgical departments, and laboratory networks of Aga Khan University Hospital (AKUH) and the Kharadar General Hospital (KGH), and surgery units of National Institute of Child Health (NICH) and Jinnah Postgraduate Medical Centre (JPMC), in Karachi, Pakistan (Figure 1). These sites were selected to include a variety of socio-economic patients, availability of patient records, and logistical access. AKUH is a 630-bed private tertiary care hospital in the metropolitan city of Karachi. The hospital serves middle- and upper-income populations; however, welfare and charity (zakat) funds are available to assist inpatients from lower-, lower-middle, and middle-income families. Being a referral hospital with state-of-the-art facilities and equipment, AKUH receives patients not only from the Sindh administrative unit, of which Karachi is the capital city but from other regions, especially from Baluchistan. The hospital maintains electronic records of both outpatient and inpatients using International Classification of Disease ninth revision (ICD-9) coding system. Each patient visiting AKUH is assigned a unique medical record number that links to all of their hospital records and is used for subsequent visits.

KGH is a 210-bed charity not-for-profit tertiary care hospital serving more than 3 million predominantly low-income people in Lyari Town, a suburb of Karachi. KGH has a paper-based medical record system where a file is maintained for each patient. From September 2016 to September 2019, all patients with suspected or confirmed typhoid were enrolled from AKUH and KGH. NICH is one of the largest public-sector children’s hospitals in Pakistan, located in Saddar Town, one of the most populated towns of Karachi. It is the largest referral center for pediatric surgery in Sindh Province. NICH has 500 pediatric beds including 2 surgical units comprising 80 beds. NICH caters to all socioeconomic levels of society, including the underprivileged majority. JPMC is a 1610 bed public sector tertiary care hospital for adults. Both NICH and JPMC have a paper-based medical record system. Enteric fever patients with intestinal perforations (surgical patients) were enrolled from NICH during November 2016-September 2019 and from JPMC during February 2018-September 2019. The AKUH laboratory network includes the main AKUH laboratory and laboratory collection points located in three secondary care hospitals in Karachi. Blood culture-confirmed enteric fever patients identified from KGH laboratory network were enrolled during December 2018–September 2019, and from the AKUH laboratory network including all collection points in Karachi enrolled from March 2019 to September 2019.

### Eligibility and Enrollment

Patients were enrolled based on location-specific eligibility criteria.

For outpatients of AKUH and KGH, enrollment in the study was self-reported history of fever for at least 3 consecutive days during the past seven days, living in the specified catchment area of the respective hospital, and blood culture advised by the physician. For inpatients at both hospitals, we enrolled both suspected and confirmed cases of enteric fever irrespective of residence.

Surgical patients with non-traumatic ileal perforations, even in the absence of laboratory confirmation, were enrolled from the surgical units of AKUH, KGH, NICH, and JPMC. Patients identified from the hospital or hospital network laboratories with blood culture positive for enteric fever were also enrolled.

### Data Collection

Trained research assistants identified and enrolled all suspected or culture-confirmed cases of enteric fever from each of the recruitment locations. If they were eligible, research assistants collected information data on socio-demographic characteristics, symptoms at the time of presentation, duration of illness, and any treatment prior to the current visit through face-to-face interviews. Data were collected on tablets with standardized forms and transfered daily to a server. Research assistants also reviewed the patient medical records to collect information on diagnosis, laboratory investigations, current treatment, complications, and duration of hospitalization Antimicrobial resistance data were obtained from the respective clinical laboratory of the sentinel hospitals. Eligible patients recruited from the laboratory networks were enrolled through telephone calls. Follow-up of all blood culture-positive patients was done by telephone six weeks after enrollment to ascertain final outcomes.

To adjust the incidence calculations from the hospital-based surveillance, we utilized a hybrid surveillance approach that involved pairing facility-based surveillance with 2 population-based healthcare utilization studies [9]: the first from January 2017 to March 2018 at the beginning of the study period, and the second from April 2018 to July 2018. The Healthcare Utilization Surveys (HCUS) were conducted in the catchment populations of both AKUH and KGH to identify the individuals in randomly selected geographical units within the study site catchment areas visiting the sentinel hospitals for treatment of enteric fever. This geographical unit census was then multiplied by the sampling probability to derive the catchment area population size [9].

**Definitions**

A laboratory-confirmed case of enteric fever was defined as a patient whose blood culture was positive for *S. Typhi* or *S. Paratyphi*. Resistance and/or intermediate resistance to ampicillin, chloramphenicol and trimethoprim-sulphamethoxazole was classified as multidrug resistant (MDR), and resistance to MDR plus fluoroquinolone and ceftriaxone was termed as extensively drug resistant (XDR).

### Data Analysis

Frequencies and percentages were calculated for the sociodemographic and clinical characteristics of enteric fever patients. *P* values were also calculated using chi square test to compare the difference between *S*. Typhi and *S.* Paratyphi patients. Similarly, frequencies and percentages were calculated for complications among resistant and sensitive cases, *P* values were calculated using Fisher exact test to compare the complications among *S*. typhi MDR/XDR cases with non MDR/XDR cases. Crude and adjusted incidence rates were estimated. Incidence rates were adjusted for (1) the sensitivity of blood culture, (2) the probability of consenting and providing blood for culture, (3) the probability of seeking care at a study site facility for individuals with suspected enteric fever, and (4) the probability of seeking care at a study site facility for individuals with suspected enteric fever accounting for differences in household education and wealth. We used a Monte Carlo approach to create distributions for each adjustment probability based on 1 million simulations and derived the median, 2.5th and 97.5th percentiles (10). The incidence rates by age group were presented with 95% confidence intervals (CIs).

### Ethical Considerations

All enrolled participants provided informed written consent. Assent was also obtained for participants 15–17 years of age. Verbal consent was obtained via telephone calls from patients enrolled from laboratory networks. The study was approved by the ethical review committee of Aga Khan University, the National Bioethics Committee (NBC) of Pakistan and Stanford University. This project was approved by the Centers for Disease Control and Prevention’s human subjects review as “research with CDC not directly engaged,” so a full IRB approval was not needed.

## RESULTS

In total, 16, 682 patients who met the eligibility criteria were approached; 11 319 from outpatients units, 4316 from inpatients units, 781 from the laboratory networks, and 266 patients from the surgery units. We enrolled, 61% (10 094/16 682) of patients screened, of whom 2,230 (22%) were blood culture positive for enteric fever: 93.9% (2,093/2,230) were culture positive for S Typhi and 6.1% (137/2,230) were positive for S. Paratyphi A (Table 1).

**Table 1.** Characteristics of culture-confirmed Enteric Fever Patients Infected by *S*. Typhi and *S*. Paratyphi, at SEAP Sites - Karachi, Pakistan, 2016–2019

Socio-demographic variables	*S.* Typhi		*S.* Paratyphi		Total		
	n = 2093	%	n = 137	%	n = 2230	%	*P-*value
Age in years							
< 2years	299	14.3	6	4.4	305	14	<.001
≥2–5 years	609	29.1	13	9.5	622	28	
≥5–15 years	784	37.5	34	24.8	818	36	
≥15–25	242	11.6	46	33.6	288	13	
≥25	159	7.6	38	27.7	197	9	
Gender							
Male	1197	57.2	89	65	1286	57.7	.074
**Year of Surveillance** ^**a**^							
September- December 2016	23	1.1	3	2.2	26	1.2	<.001
2017	281	13.4	87	63.5	368	16.5	
2018	794	37.9	26	19	820	36.8	
January- September2019	995	47.5	21	15.3	1016	45.6	
**Recruitment Location**							
(Enrolled/Screened) ^b^							
Outpatient	724 (724/11 319)	34.6	24 (24/11 319)	17.5	748	33.5	<.001
					(748/11 319)		
Inpatient	629 (629/4316)	30.1	65 (65/4316)	47.5	694 (694/4316)	31.1	
Hospital laboratory and laboratory network	732	35	48	35	780	35	
Surgery	8 (8/266)	0.4	0 (0/266)	0	8 (8/266)	0.4	
**Socio-economic status**							
Low SES	638/1541	41	16/119	13	654/1660	39	<.001
Middle SES	317/1541	21	20/119	17	337/1660	20	
High SES	556/1541	36	82/119	69	638/1660	38	
**Lost to Follow-up**	30/1541	2	1/119	1	31/1660	2	<.001
**Districts**							
East	647	30.9	94	68.6	741	33.2	<.001
Central	213	10.2	15	11	228	10.2	
South	862	41.2	13	9.5	875	39.2	
West	149	7.1	2	1.5	151	6.8	
Others (Out of Karachi)	222	10.6	13	9.5	235	10.5	
**Patient sought any care before visiting hospital** ^**c**^							
Pharmacy	149	7.1	15	10.9	164	7.4	.059
Clinic	572	27.3	39	28.5	611	27.4	.29
Physician	563	26.9	21	15.3	584	26.2	.04
Others # (Traditional Healer/Lab/Self Medication)	58	2.8	0	0	58	2.6	.048
MDR cases among tested	331/2084	15.9	3/135	2.2	334/2219	15.1	<.001
XDR cases among tested	1319/2077	63.5	0/134	0	1319/2211	59.7	<.001
Duration of illness (time to seeking care)							-
Median (IQR)	8 (5, 13)		8 (5, 14)		8 (5, 13)		
**Final Outcome of patients**	**n = 855**	**%**	**n = 35**	**%**	**n = 890**	**%**	
Discharged	834	97.5	34	97.1	868	97.5	.91
Referred to another hospital	1	0.1	0	0	1	0.1	
Left against medical advice	11	1.3	1	2.9	12	1.4	
Died	4	0.5	0	0	4	0.4	
Withdrew consent	6	0.7	0	0	6	0.7	

^a^Data collection for year 2016 was only from September to December 2016.

^b^Numbers of enrolled out of screened (for hospital laboratory and laboratory network only culture confirmed cases were enrolled therefore not screened).

^c^Percentages don’t add as 100% due to multiple answers, # (Home remedies, Community Health Workers CHW).

**Figure 2.**
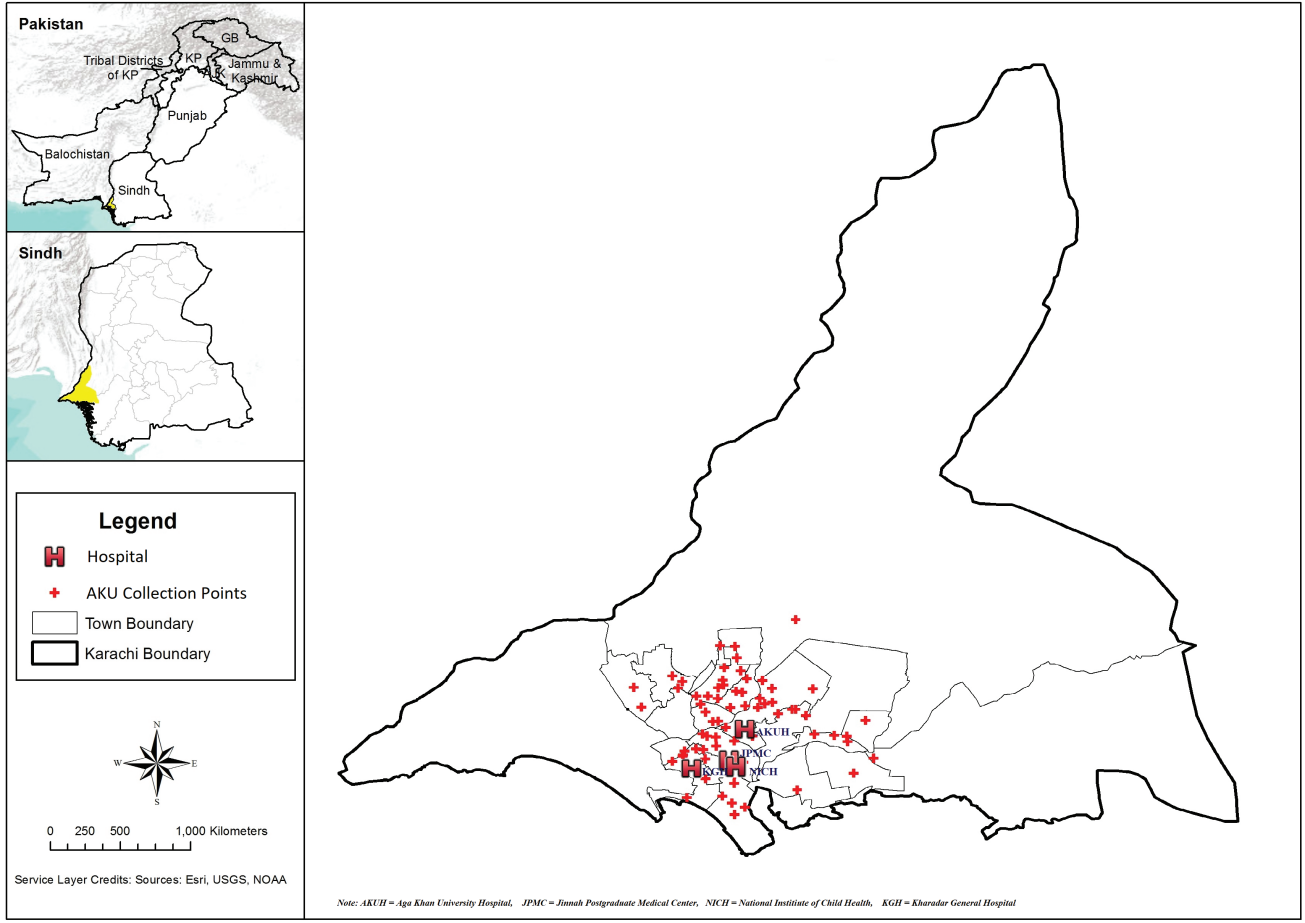
Monthly trends of culture-confirmed enteric fever cases, Karachi, Pakistan, 2016–2019.

Overall, 58% (1,286/2,230) of the culture-confirmed cases were male; 78% (1,745/2,230) were children aged <15 years old; and among 1,660 cases for whom information was available, 991 (60%) were of lower or middle SES. Approximately one-third of culture-confirmed cases were recruited from each of the outpatient, inpatient and laboratory settings. The highest number of culture-confirmed enteric fever cases 1,016 (46%) were observed in year 2019 (Table 1), with seasonal peaks from April to June (Figure 2). Overall, 15% (334/2,219) of the culture-confirmed enteric fever patients who had antimicrobial susceptibility testing results available had isolates that were resistant to first line antibiotics (MDR), and 60% (1,319/2,211) of culture-confirmed typhoid cases had isolates that were XDR (Table 1).The total adjusted incidence rate for culture-confirmed S. Typhi cases was 127 per 100,000 person years at AKUH and 195 per 100,000 at KGH; for children aged 6 months–15years, the incidence rate was 371 per 100,000 at AKUH and 527 per 100, 000 at KGH (Table 2). The adjusted incidence rates were highest among S. Typhi aged 2 to 4 years (481 per 100,000 from AKUH and 1,009 per 100,000 from KGH), and lowest among patients >25 years old (26 per 100,000 from AKUH and 9 per 100,000 from KGH). There was no difference in the incidence rates by hospitals (Table 2). The results from the healthcare utilization survey, accounting for one adjustment, are presented elsewhere in this supplement Andrews et al paper that begins on S248.

**Table 2.** Crude and Adjusted Incidence Rates by Age among culture-confirmed enteric fever cases at SEAP Study Hospitals - Karachi, Pakistan 2016–2019

	AKUH		AKUH		KGH		KGH	
	*S.* Typhi		*S*. Paratyphi		*S.* Typhi		*S*. Paratyphi	
Age Group	CR^a^(95% CI)	AIR^b^ (95% CI)	CR^a^(95% CI)	AIR^b^ (95% CI)	CR^a^(95% CI)	AIR^b^ (95% CI)	CR^a^(95% CI)	AIR^b^ (95% CI)
**<2 years**	43 (24, 74)	373 (258, 559)	1(0, 12)	10 (6, 18)	175 (122, 244)	658 (499, 920)	4 (0, 28)	8 (6, 12)
**2 to 4 years**	36 (24, 54)	481 (360, 655)	4 (0, 10)	38 (26, 56)	146 (113, 185)	1009 (762, 1383)	1 (0, 8)	6 (4, 8)
**5 to 15 years**	25 (19, 32)	361 (273, 485)	3 (2, 7)	48 (35, 66)	29 (21, 38)	378 (282, 519)	0 (0, 2)	-
**16 to 25 years**	11 (7, 17)	177 (126, 256)	4 (2, 8)	69 (49, 100)	3 (1, 8)	43 (30, 64)	-	-
**>25 years**	3 (2, 5)	26 (21, 32)	2 (1, 3)	11 (9, 14)	1(0, 3)	9 (7, 12)	-	-
**6 months to 15 years**	27 (22, 33)	371 (297, 468)	3 (2, 6)	39 (30, 51)	66 (56, 78)	527 (428, 660)	0.3 (0, 2.4)	3 (2, 3)
**Total**	12 (10, 14)	127 (106, 151)	3 (2, 4)	25 (21, 31)	24 (21, 28)	195 (163, 236)	0.1 (0, .8)	1 (1)

^a^CR = Crude rate per 100 000.

^b^AIR = Adjusted Incidence rate per 100 000.

*(Incidence rates were adjusted for* (1) *the sensitivity of blood culture* (2)*, the probability of consenting and providing blood for culture* (3)*, the probability of seeking care at a study site facility for individuals with suspected enteric fever, and* (4) *the probability of seeking care at a study site facility for individuals with suspected enteric fever accounting for differences in household education and wealth).*

Before visiting the respective sentinel hospital, 27.4% of the patients visited a nearby first-level care facility/clinic (Table 1). At the time of the hospital visit, 97% (2171/2230) of patients presented with fever, followed by vomiting 53% (1189/2230) and abdominal pain 43% (961/2230). Of the culture confirmed Paratyphi patients, 66% (90/137) presented with headache. Moreover, 58% (1292/2230) of patients reported taking antibiotics before visiting the sentinel hospital (Table 3). There were four deaths among culture-confirmed cases of enteric fever with a mortality rate of 0.4% (4/10,094).

**Table 3.** Clinical Characteristics of Enteric Fever Patients Infected by *S*. Typhi and *S*. Paratyphi in SEAP Sites - Karachi, Pakistan, 2016–2019.

	*S.* Typhi		*S.* Paratyphi		Total		
Clinical characteristics	n = 2093	%	n = 137	%	n = 2230	%	*P*-value
**Symptoms at the time of presentation**							
Fever	2037	97.3	134	97.8	2171	97.4	.69
Vomiting	1125	53.8	64	46.7	1189	53.3	.29
Abdominal pain	905	43.2	56	40.9	961	43.1	.68
Cough	759	36.3	61	44.5	820	36.8	.13
Headache	717	34.3	90	65.7	807	36.2	<.001
Diarrhea	617	29.5	35	25.5	652	29.2	<.001
Difficulty breathing	253	12.1	25	18.2	278	12.5	.04
Confusion	192	9.2	14	10.2	206	9.2	.001
Constipation	118	5.6	7	5.1	125	5.6	.001
Rash	54	2.6	6	4.4	60	2.7	.36
Seizure	16	0.8	1	0.7	17	0.8	<.001
Jaundice	49	2.3	2	1.5	51	2.3	.14
Blood in stool	25	1.2	0	0	25	1.1	.25
Loss of appetite	757	36.2	19	13.9	776	34.8	<.001
Generalized weakness	489	23.4	34	24.8	523	23.5	.69
Lower urinary tract symptom	20	1.0	0	0	20	0.9	.25
Patient reported taking antibiotic prior to enrollment visit	1216	58.1	76	55.5	1292	57.9	.65
Patient reported taking antipyretics prior to enrollment visit	1796	85.8	132	96.4	1928	86.5	<.001

Complications such as hepatitis, hemodynamic shock, pulmonary complications, gastrointestinal complications, and sepsis were more frequently identified among typhoid patients with XDR and MDR *S*. Typhi as compared to non-XDR/MDR patients of typhoid [10].

## DISCUSSION

Enteric fever is endemic in Karachi, Pakistan, with the highest incidence in children < 15 years, which is similar to previous studies from Bangladesh and India [11, 12]. Overall, the incidence was highest among patients presenting to KGH. We report a higher number of culture-confirmed cases of typhoid in males. Similar findings have also been reported from other endemic settings [13–16]. While no biological explanation is available in the literature for the preponderance of *S.* Typhi among males, the most plausible reasons could be relatively higher outdoor exposure, eating out, and behaviors, and attitudes of male population resulting in increased risk for *S.* Typhi infections in developing countries [16]. Moreover, greater healthcare seeking by parents of male children could be another reason [13].

Although an annual monsoon season does not occur in Karachi, we noted a distinct seasonal variation, with most of the reported cases occurring in the summer season, which does receive some rainfall. The highest numbers of cases were consistently reported in April to September, while December to February showed a comparably low caseload during the 3 years. In congruence with seasonality patterns elsewhere, similar findings have been presented in previous studies from Karachi that showed a high incidence in the summer months [17]. Environmental factors such as rainfall may have a substantial influence on the occurrence of typhoid [18, 19] with increasing transmission of waterborne pathogens during wet periods [20].

**Figure 3.**
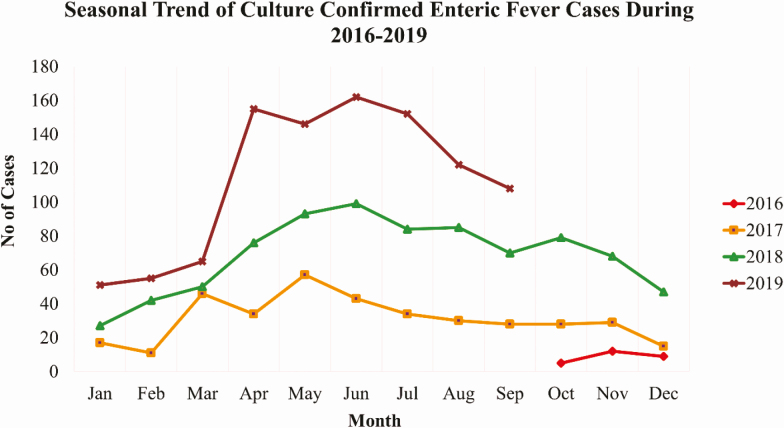
Yearly trends of extensively drug-resistant enteric fever cases, Karachi, Pakistan, 2016–2019. Abbreviation: XDR, extensively drug-resistant.

During the study period, we identified a low proportion of deaths among all confirmed cases. A previous study from Pakistan reported similar mortality attributed to typhoid [21]. Studies from other endemic countries report substantial variation in mortality. A study in Vietnam reported 2% mortality attributed to *S.* Typhi among hospitalized patients [22]. Age-related differences in enteric fever mortality also vary globally. All fatal cases in our study were children aged 1 to 8 years, with none attributed to paratyphoid fever, but due to complications, including septic shock and multiorgan failure, pulmonary complications, and cardiac arrest. Complications among MDR and XDR cases were more frequent as compared to the non MDR/non XDR cases. Mortality was low in our study, but also highest among all of the SEAP sites, and all deaths were associated with antimicrobial-resistant strains during the study period. A rising trend of MDR and XDR strains of *S.* Typhi was observed during the study period (Figure 3). This increase in antimicrobial resistance in *S.* Typhi requires an immediate response at the global level. As with a few antibiotic choices left for the effective treatment of *S.* Typhi, not only mortality and complications will increase but the cost of treatment will also increase many fold.

The results of this study highlight the importance of vaccination and other preventive measures in this population. In 2018, the World Health Organization (WHO) has prequalified and recommended a single dose of typhoid conjugate vaccine (TCV) among children living in countries where typhoid is endemic and among travelers visiting these countries [23]. The evidence generated through the ongoing SEAP surveillance has helped the government of Pakistan to apply for support from GAVI, the Vaccine Alliance, for the introduction of TCV in the routine immunization program in Pakistan. In the last quarter of 2019, a mass TCV immunization campaign was conducted for children aged 9 months to 15 years of age in Sindh province, which also includes the capital Karachi, with the plan of introducing a single dose of TCV at 9 months of age in routine immunization from 2020. With sufficient coverage, TCV is hoped to reduce the burden of typhoid in Pakistan.

Vaccination might also be effective in reducing the antimicrobial resistance against *S.* Typhi. Previous studies from the introduction of pneumococcal conjugate vaccine (PCV) and Hemophilus influenza type B (HiB) have demonstrated that in some contexts, vaccines can reduce illnesses due to antimicrobial-resistant strains [24, 25]. Furthermore, in typhoid endemic countries such as Pakistan, the majority of cases of febrile illness of more than 3 days receive an antibiotic without confirmation of diagnosis through blood culture [26] due to lack of availability of accurate diagnostics, cost of blood culture, and feasibility [27]. With the expected decrease in the burden of typhoid after the catch-up vaccination campaign in November 2019 to December 2019, prescriptions for antibiotics should decrease with time.

While vaccination is an effective and cost-efficient short-term solution to decrease the burden of *S.* Typhi in Pakistan, sustainable long-term solutions lie in the provision of clean drinking water, a sanitation system that minimizes fecal environmental contamination, and avoiding the use of contaminated food. As one of the United Nations Sustainable Development Goals, the provision of clean drinking water to the general population in each of the signatory countries is an essential aspect to the living and wellbeing of the masses.

A strength of this prospective surveillance study was the use of a standard case definition for participant enrollment and blood culture confirmation. The use of hybrid surveillance, which has been employed for several other infectious diseases, enabled the generation of population-based occurrence estimates with considerably fewer resources than cohort studies [28, 29]. The study included cases from the 2 tertiary care hospitals, located in urban areas and laboratory networks of Karachi, which cater to all parts of the city of Karachi (Figure 1). The results of the study can therefore be generalized to the urban sites of country.

Our study had several limitations. Hospital-based surveillance tends to capture only the severe form of the disease and could miss less severe cases of the disease. Although we included multiple surveillance sites in our study, these were in the metropolitan city of Karachi, Pakistan, an urban setting that prevents our incidence estimates to be generalized to the rural population of Karachi or elsewhere in Pakistan. The sensitivity of blood culture testing for enteric fever was sub-optimum and hence some cases of typhoid and paratyphoid might have been missed or misclassified, which likely led to underreporting of cases.

## CONCLUSIONS

In summary, enteric fever is common in Karachi, Pakistan. The trend of culture-confirmed enteric fever, especially *S.* Typhi, has increased over the surveillance period. Antimicrobial resistance also increased over the study period, with the emergence of XDR typhoid. The burden of paratyphoid is still relatively smaller, however, strengthening the existing surveillance system at the national level for both typhoid and paratyphoid and is recommended in Pakistan. While TCV vaccination can significantly decrease the burden of typhoid and may also impact antimicrobial resistance, investments in long-term sustainable interventions such as clean drinking water, environmental hygiene, and sanitation is highly recommended.

## Notes

***Financial support***. This work was supported by the Bill & Melinda Gates Foundation [Investment ID 008335].

***Supplement sponsorship.*** This supplement is sponsored by the Sabin Vaccine Institute and made possible by a grant from the Bill & Melinda Gates Foundation.

***Potential conflicts of interest***. S. S. reports grants from the World Health Organization during the conduct of the study; and grants from the World Health Organization, Bill & Melinda Gates Foundation, GlaxoSmithKline, Pfizer, Sanofi Pasteur, and Edinburgh University, outside the submitted work. All other authors report no potential conflicts. All authors have submitted the ICMJE Form for Disclosure of Potential Conflicts of Interest. Conflicts that the editors consider relevant to the content of the manuscript have been disclosed.
